# Insulin resistance induced by obesity: Mechanisms, metabolic implications and therapeutic approaches

**DOI:** 10.1007/s11033-026-11509-3

**Published:** 2026-02-04

**Authors:** Yi Ning Choo, Ram Narayanan Ravi, Vetriselvan Subramaniyan

**Affiliations:** 1 Sir Jeffrey Cheah Sunway Medical School, Faculty of Medical and Life Sciences, Sunway University, Bandar, Malaysia; 2 Jeffrey Cheah School of Medicine and Health Sciences, Monash University Malaysia, Bandar Sunway Selangor Darul Ehsan, Bandar, Malaysia

**Keywords:** Insulin resistance, Inflammation, Oxidative stress, Personalized medicine

## Abstract

Obesity-induced insulin resistance is an escalating global health challenge that substantially contributes to the development of metabolic disorders, including type 2 diabetes mellitus and cardiovascular disease. This narrative review critically examines the molecular and cellular mechanisms linking excess adiposity to impaired insulin action, with a particular focus on adipose tissue dysfunction, chronic low-grade inflammation, and oxidative stress. A comprehensive literature search was conducted using PubMed, ScienceDirect, and Google Scholar, covering preclinical and clinical studies published primarily over the past two decades. Evidence indicates that adipocyte hypertrophy and hypoxia promote excessive free fatty acid release, ectopic lipid accumulation, and lipotoxicity, thereby disrupting insulin signalling pathways. Numerous clinical studies report that obesity triggers chronic, low-grade inflammation in the liver and pancreas, activating pathways such as NF-κB and SOCS proteins, thereby disrupting insulin signalling. Concurrently, obesity-associated inflammation drives immune cell infiltration and macrophage polarization toward a pro-inflammatory phenotype, further exacerbating insulin resistance and metabolic dysregulation. This review also synthesizes current therapeutic strategies targeting these mechanisms, including insulin-sensitizing agents, anti-inflammatory therapies, glucagon-like peptide-1 receptor agonists, and sodium-glucose cotransporter 2 inhibitors, as well as emerging approaches. Future perspectives highlight the growing relevance of personalized medicine, pharmacogenomics, digital health tools, and gene-based interventions in improving therapeutic precision. A deeper understanding of these interconnected pathways is essential for developing effective strategies to mitigate obesity-related insulin resistance and its global metabolic consequences.

**Graphical Abstract:**

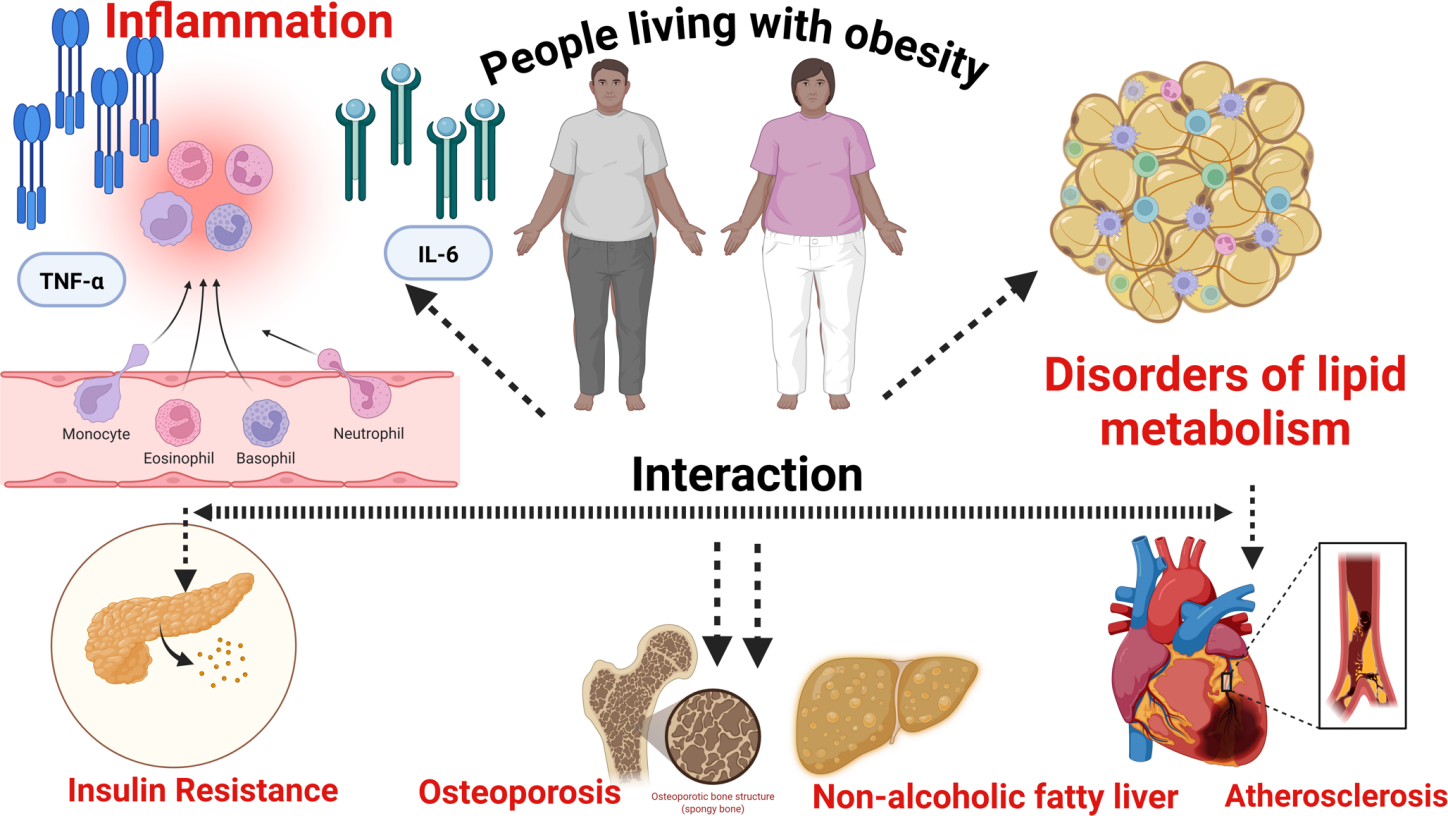

## Introduction

Obesity is the excessive accumulation of adipose tissue due to increased nutrient intake and insufficient energy expenditure [1]. A body mass index (BMI) greater than 25 is considered overweight, and greater than 30 Kg/m^2^ is obese [2]. According to the World Health Organization, around 5 million non-communicable disease (NCD) deaths were caused by high BMI [3]. Insulin is a hormone secreted by β-cells of the islets of Langerhans [4]. It regulates energy storage and glucose metabolism in the body by taking up glucose from the blood and converting it into glycogen to store in muscle and liver [5]. Insulin resistance is the condition in which the responsiveness of insulin-targeting tissues is reduced although the insulin is present at high physiological levels [6]. Excessive fat accumulation in obesity can pose significant health risks, including insulin resistance. In obesity, adipose tissue becomes dysfunctional, leading to adipose hypertrophy and adipose hypoxia, which impact the normal function of adipose tissue. This causes an elevated amount of non-esterified fatty acids, glycerol and pro-inflammatory cytokines, which contribute to the development of insulin resistance by affecting insulin signalling pathway, lipid and glucose metabolism [7]. Excessive free fatty acids and lipids accumulate in non-adipose tissues such as the liver and muscle, leading to lipotoxicity [8]. This disrupts cellular organelles and contributes to insulin resistance. Besides, obesity can induce chronic low-grade inflammation and oxidative stress, which are critical in the pathogenesis of insulin resistance [9].

**Fig. 1:**
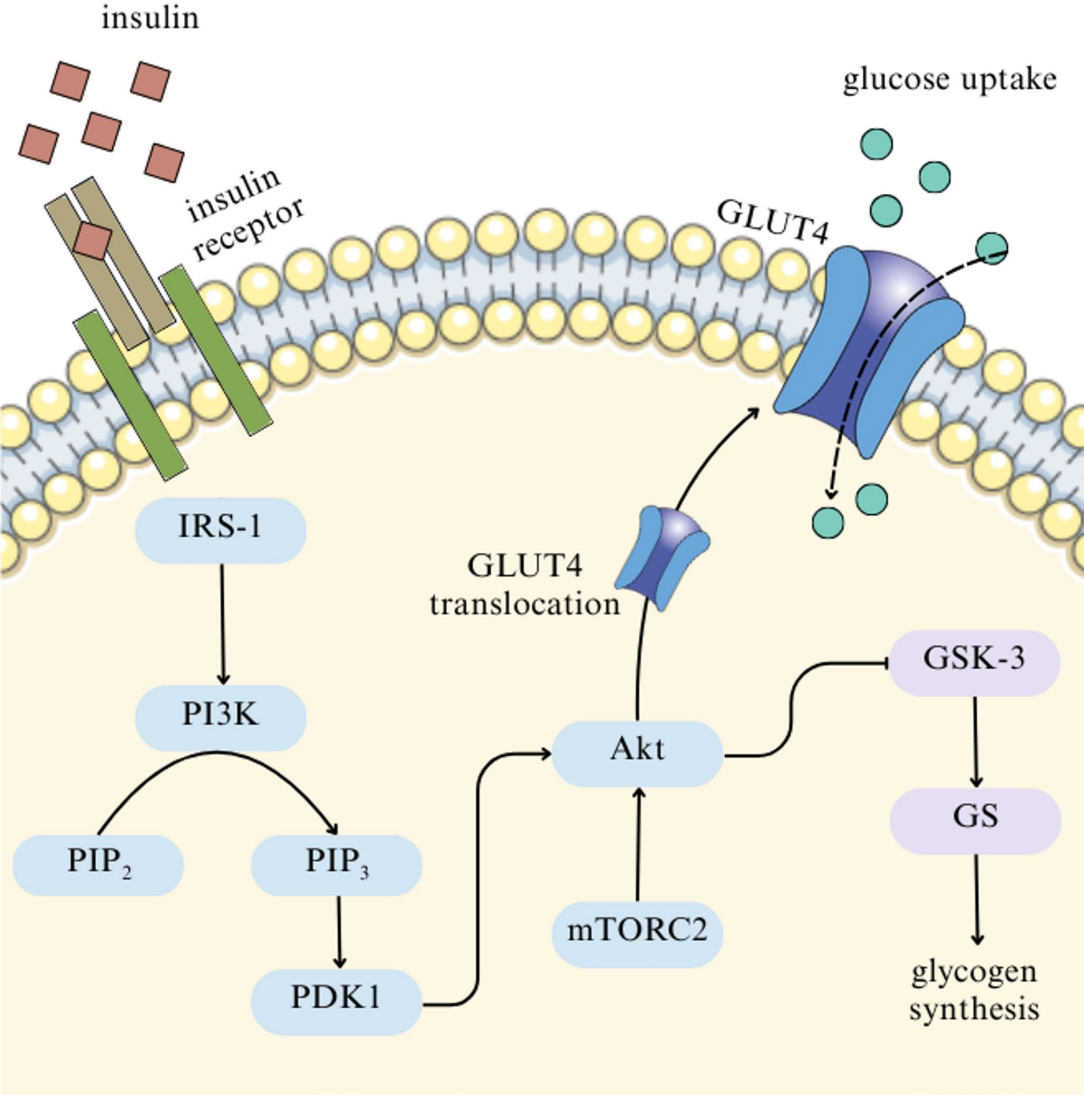
Schematic representation of insulin signalling pathway. Insulin binds to the insulin receptor, leading to activation of IRS-1 and PI3K. PI3K catalyzes the conversion of PIP2 to PIP3, which activates PDK1. PDK1, together with mTORC2, phosphorylates and activates Akt. Activated Akt promotes GLUT4 translocation to the plasma membrane, enhancing glucose uptake, and inhibits GSK-3, thereby stimulating glycogen synthase activity and glycogen synthesis. IRS-1, insulin receptor substrate 1; PI3K, phosphatidylinositol 3-kinase; PIP2, phosphatidylinositol 4,5-bisphosphate; PIP3, phosphatidylinositol 3,4,5-trisphosphate; PDK1, phosphoinositide-dependent kinase 1; mTORC2, mechanistic target of rapamycin complex 2; Akt, protein kinase B; GLUT4, glucose transporter 4; GSK-3, glycogen synthase kinase-3. Illustration created using Canva (https://www.canva.com)

The inflammation is often due to macrophage infiltration, and the release of pro-inflammatory cytokines and inhibit anti-inflammatory cytokines in adipose tissue. Obesity-induced chronic low-grade inflammation and oxidative stress potentially alter the normal insulin signalling pathway. In normal conditions, insulin binds to its receptor, activating downstream pathways, such as the phosphatidylinositol 3-kinase (PI3K) pathway, which facilitates glucose uptake and metabolism [10]. Insulin binds to the α-subunit of the insulin receptor, leading to the self-phosphorylation of tyrosine residues on the β-subunit [11]. Once the insulin receptor is activated, it triggers the phosphorylation of insulin receptor substrate 1 (IRS-1). The activated IRS-1 recruits PI3K [12]. PI3K converts phosphatidylinositol 4,5-bisphosphate (PIP₂) to phosphatidylinositol 3,4,5-trisphosphate (PIP₃). PIP₃ recruits phosphoinositide-dependent kinase 1 (PDK1). PDK1 [13, 14] phosphorylates Akt at Thr^308^; at the same time, the mechanistic target of rapamycin complex 2 (mTORC2) phosphorylates Akt at Ser^473^. Both phosphorylation activates the protein kinase B (Akt) pathway. Akt triggers the translocation of glucose transporter 4 (GLUT4), promoting glucose uptake from the bloodstream into cells [15]. Akt also inhibits the activity of glycogen synthase kinase-3 (GSK-3). Inactive GSK-3 allows the activity of glycogen synthase (GS), which promotes glycogen synthesis [16]. The normal insulin signalling pathway is summarized in Fig. 1.

However, in obese condition, the inflammation and oxidative stress could activate inflammatory pathways, such as inhibitory kappa B kinase beta (IKKβ) and c-Jun N-terminal kinase (JNK) [17]. These pathways can deactivate PI3-K pathway and disrupt the downstream pathways. As a result, glucose uptake reduced in the condition where insulin is sufficient, thus inducing insulin resistance. Although numerous reviews have summarized obesity-induced insulin resistance, most focus on individual treatment approaches or overarching causes. The central hypothesis of this paper is that obesity-induced insulin resistance arises from integrated adipose tissue dysfunction, inflammation, and oxidative stress, impairing insulin signalling. This review fills the gap by offering a thorough synthesis of conventional medications, natural substances, lifestyle modifications, and innovative treatments, emphasizing recent molecular discoveries and translational prospects. The aim of this narrative review is to critically synthesize current preclinical and clinical evidence on the pathophysiological mechanisms underlying obesity-induced insulin resistance, with particular emphasis on adipose tissue dysfunction, chronic inflammation, and oxidative stress, and to evaluate established and emerging therapeutic approaches that target these mechanisms to restore insulin sensitivity and reduce metabolic complications.

## Materials and methods

The inclusion criteria were based on the PICOS (Population, Intervention, Comparators, Outcomes, Study Design) format. A systematic search was conducted on PubMed, ScienceDirect and Google Scholar databases for relevant studies (Clinical and pre-clinical) covering the timeframe of 2000 to 2025. Search terms included “insulin resistance AND obesity,” “insulin AND obesity AND pediatrics OR children OR pediatrics,” “therapeutic approaches AND obesity AND adults,” and “Novel treatment strategies AND obesity and pediatrics OR children OR pediatrics.” This review aims to close the identified gap by addressing the following inquiries:

### RQ1

What molecular and cellular mechanisms link obesity to insulin resistance?

### RQ2

What therapeutic strategies are supported by current preclinical and clinical evidence to mitigate obesity-induced insulin resistance?

### The role of dysfunctional adipose tissue in metabolic dysregulation

This section discusses adipocyte hypertrophy, hypoxia, adipokine imbalance, ER stress, and ectopic fat accumulation, while excluding unrelated adipose biology.

#### Adipocyte hypertrophy

Dysfunctional adipose tissue in people living with obesity, such as adipocyte hypertrophy and hypoxia, can cause a series of metabolic dysregulations and lead to insulin resistance. Adipocyte hypertrophy is the enlargement of adipose tissue [18]. It is an adaptive response in which adipose tissue serves as a nutrient-buffering capacity when nutrients are in excess, while protecting other tissues from lipotoxicity [19]. However, in an obese state, the hypertrophic adipose tissue is beyond the threshold, exceeding its buffering capacity. Adipocyte hypertrophy increases lipolysis [20]. Excessive lipolysis releases large amounts of free fatty acids (FFAs) into the bloodstream [21]. This may lead to ectopic fat deposition, which is the accumulation of excessive fat in non-adipose tissue, such as the liver, skeletal muscle, heart and pancreas [22, 23]. Ectopic fat serves as a dysfunctional adipose tissue that causes lipotoxicity and may further disrupt the normal metabolic function of the adipocyte, leading to the development of insulin resistance [24, 25]. Adipocyte hypertrophy may lead to insulin resistance by inducing inflammation and oxidative stress, altering the insulin signalling pathway, disrupting the normal adipocyte function, and modifying gene expression. The enlargement of adipose tissue induces endoplasmic reticulum (ER) stress [26]. This triggers the inflammatory pathway by releasing the pro-inflammatory cytokines, including tumour necrosis factor α (TNF-α) and interleukin-6 (IL-6) [27]. In addition to the release of cytokines adipocyte hypertrophy triggers a complex inflammatory cascade by secreting Serum Amyloid A (SAA) to recruit macrophages and Complement Factor H to activate the alternative complement pathway. Simultaneously, dysregulated Aquaporins (AQP7 and AQP11) impair glycerol transport and promote oxidative stress, further fueling cell enlargement and systemic metabolic dysfunction [28–30].

**Fig. 2:**
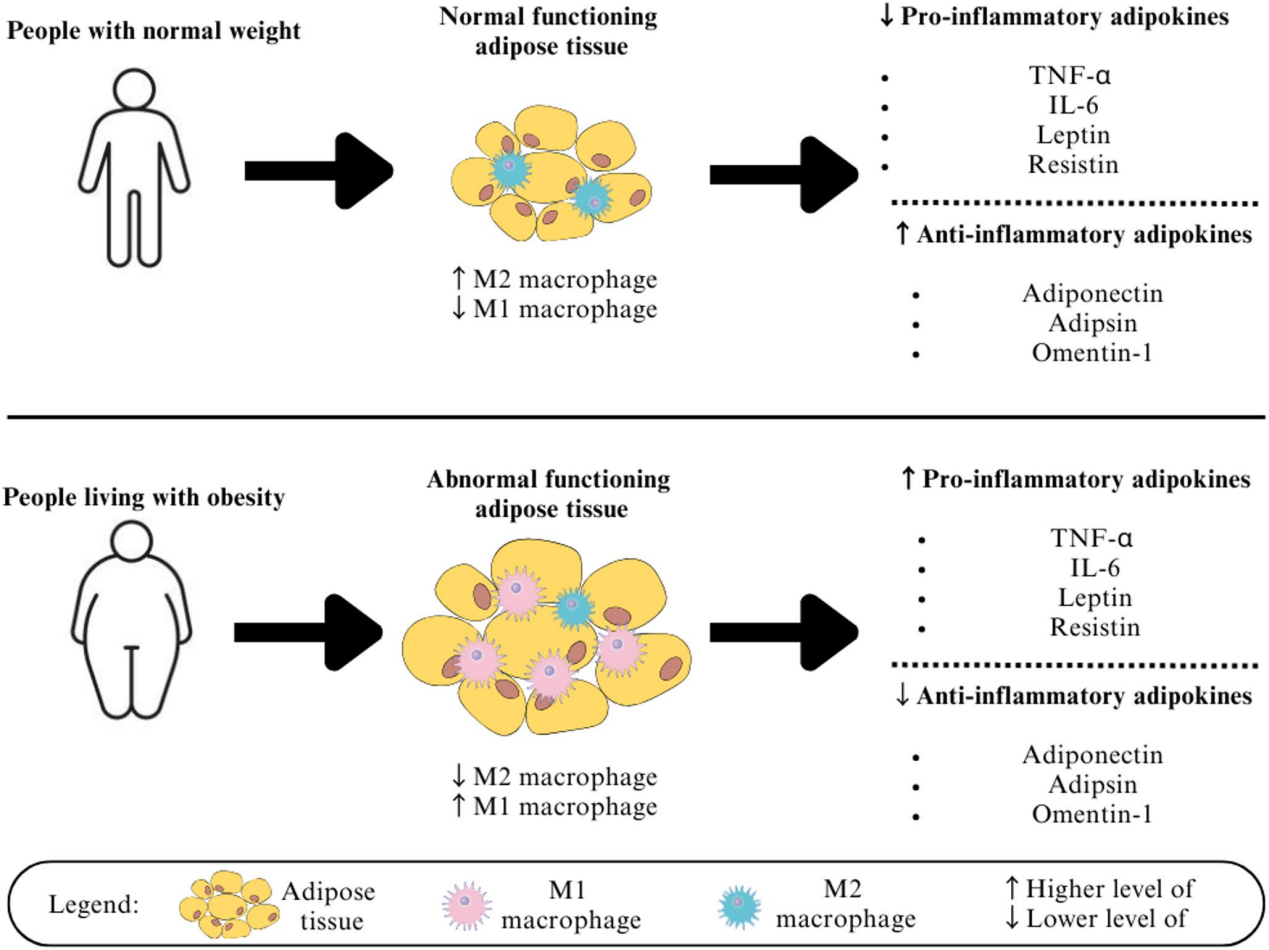
Comparison of adipokine levels between normal individuals and individuals with obesity. In normal individuals, adipose tissue is characterized by a higher proportion of anti-inflammatory M2 macrophages and a lower proportion of pro-inflammatory M1 macrophages, resulting in reduced secretion of pro-inflammatory adipokines such as TNF-α, IL-6, leptin, and resistin, along with increased secretion of anti-inflammatory adipokines including adiponectin, adipsin, and omentin-1. In contrast, individuals with obesity exhibit adipose tissue dysfunction characterized by increased M1 macrophage infiltration, reduced M2 macrophage numbers, elevated levels of pro-inflammatory adipokines, and decreased production of anti-inflammatory adipokines, contributing to chronic low-grade inflammation and insulin resistance. TNF-α, tumor necrosis factor alpha; IL-6, interleukin-6. Illustration created using Canva (https://www.canva.com)

Elevated levels of such cytokines alter insulin signal transduction by inducing inflammation, thereby affecting glucose uptake, decreasing insulin sensitivity, and leading to insulin resistance [31]. As the adipocytes expand, dysregulated adipokine secretion may occur. Adipose tissue is triggered to secrete more pro-inflammatory and insulin-resistant adipokines such as TNF-α, IL-6, resistin and leptin [32–34]. Leptin is reported to be involved in elevation of cardiovascular risk factors and one of the important predictor of circulating fibrinogen concentrations [35]. Simultaneously, the adipose tissue will secrete less anti-inflammatory and insulin-sensitizing adipokines such as adiponectin, adipsin and omentin-1. Adipokine imbalances contribute to the development of insulin resistance. As shown in Fig. 2, the comparison of adipokine levels between normal individuals and individuals with obesity. Moreover, the hypertrophic state of adipose tissue upregulated exosomal miR802-5p, which targets heat shock protein 60 (HSP60) [36]. Silencing of HSP60 induces oxidative stress and impairs insulin signalling. This reduces insulin sensitivity and causes insulin resistance in cardiac myocytes.

#### Adipocyte hypoxia

As adipose tissue cell size increases, it consumes more oxygen [37]. A previous study reported that hypertrophic adipocytes can reach a diameter of 140–180 μm [27]. However, the diffusion limit of oxygen is typically 100 μm [38]. When adipocyte diameter increases, the distance between adipocytes and blood vessels increases, thereby reducing oxygen tension and creating a low-oxygen availability region [39]. If the existing blood supply cannot keep pace with increased oxygen demand, the hypertrophic adipocyte will be in an oxygen-deficient state, called adipocyte hypoxia [40]. Adipocyte hypoxia will activate the hypoxia-inducible factor-1α (HIF-1α) in adipose tissue [41]. The expression of HIF-1α then promotes inflammation and induces adipokine imbalance [37, 39]. This disrupts insulin signalling and glucose uptake, leading to insulin resistance. Associated with adipocyte necrosis and inflammation caused by adipocyte hypoxia, inflammatory cytokines such as TNF-α are released, impairing insulin signaling [42].

### Mechanisms of inflammatory and oxidative stress in insulin resistance associated with obesity

This section focuses on pathways directly relevant to obesity-driven insulin resistance, particularly NF-κB, IKKβ, and JNK signalling.

### Chronic Low-grade inflammation

Chronic low-grade inflammation and oxidative stress are the two main obesity-related metabolic consequences strongly linked to insulin resistance. As mentioned above, both adipocyte hypertrophy and hypoxia can cause chronic low-grade inflammation. In an obese condition, adipose tissue accumulates more macrophages [43]. Macrophages tend to polarize, changing their phenotype from M2 to M1 [44]. This process is triggered by pro-inflammatory signals from cytokines, high levels of FFAs, and chemokines produced by adipose tissue in people living with obesity, which activate Toll-like receptor 4 (TLR4) on macrophages [45]. Initially, in the M2 phenotype, macrophages exhibit anti-inflammatory properties, secreting cytokines such as interleukin-10 (IL-10), transforming growth factor beta 1 (TGF-β1), and arginase-1. However, when the macrophages shift to the M1 phenotype, they exhibit pro-inflammatory properties, secreting pro-inflammatory cytokines such as TNF-α and IL-6, activating the downstream pathway and leading to insulin resistance [46].

Recent investigations [47] indicate that exosomal microRNAs from hypertrophic adipocytes, such as miR-802-5p, directly disrupt insulin signalling in peripheral organs, elucidating the mechanisms behind obesity-induced insulin resistance. Furthermore, recent findings from CRISPR-mediated gene editing of NRIP1 and FABP4 in mouse models underscore possible mechanisms to restore insulin resistance, hence endorsing tailored therapy approaches.

### Oxidative stress

Oxidative stress is caused by the imbalance between decreased antioxidant defences and the overproduction of reactive oxygen species (ROS) [48]. The increased ROS levels in people living with obesity result from mitochondrial dysfunction and the activation of nicotinamide adenine dinucleotide phosphate (NADPH) oxidase (NOX) driven by excess nutrients and inflammation. About 90% of cellular ROS is generated by mitochondria [49]. They are primarily produced through oxidative phosphorylation at the electron transport chain (ETC). Electrons escape from Complex I and III in the ETC and interact with molecular oxygen to form superoxide radicals, which are one of the ROS [50]. The accumulation of superoxide causes oxidative damage to mitochondria, further disrupting their normal function and producing more ROS [51]. Furthermore, the NOX enzyme is stimulated by excessive FFAs in adipose tissue [52]. For instance, NOX4 is one of the NOX enzymes that generate hydrogen peroxide [53]. The ROS generated will then activate the downstream pathway, contributing to insulin resistance.

### Activation of nuclear Factor-kappa B (NF-κB) pathway

The nuclear factor-kappa B (NF-κB) pathway is the primary pathway related to insulin resistance [17]. It is activated by hypoxia, ER stress, ROS, FFAs and inflammatory cytokines (TNF-α and IL-1β) [54, 55]. Adipocyte hypoxia activates HIF-1α, which increases the expression of the Receptor for Advanced Glycation End Products (RAGE) [56]. RAGE then activates the NF-κB pathway. When ER stress exists, the Unfolded Protein Response (UPR) will be activated via transmembrane proteins, inositol-requiring enzyme 1 (IRE1), protein kinase RNA-like ER kinase (PERK) and activating transcription factor 6 (ATF6) [57]. Once IRE1 is activated, it recruits tumour necrosis factor receptor-associated factor 2 (TRAF2) to signal to the IκB kinase (IKK) complex. The complex activates the NF-κB signalling pathway to induce an inflammatory response. ROS contributes to oxidative stress. This condition activates IKK through oxidative modification of cysteine residues in the IKK complex [58]. Activated IKK complex then facilitates the NF-κB pathway. FFAs can activate the NF-κB pathway via toll-like receptor (TLR) ligation. They interact with TLR2 and TLR4, then recruit myeloid differentiation primary response 88 (MyD88), activating the IKK complex and thus activating the NF-κB pathway for inflammation [59]. In addition, inflammatory cytokines act as ligands that bind their receptors, initiating the NF-κB pathway. For instance, TNF-α binds to tumour necrosis factor receptor 1 (TNFR1) to activate the IKK complex and thus activate NF-κB pathway [60].

### Mechanism of nuclear factor-kappa B (NF-κB) pathway in insulin resistance

**Fig. 3:**
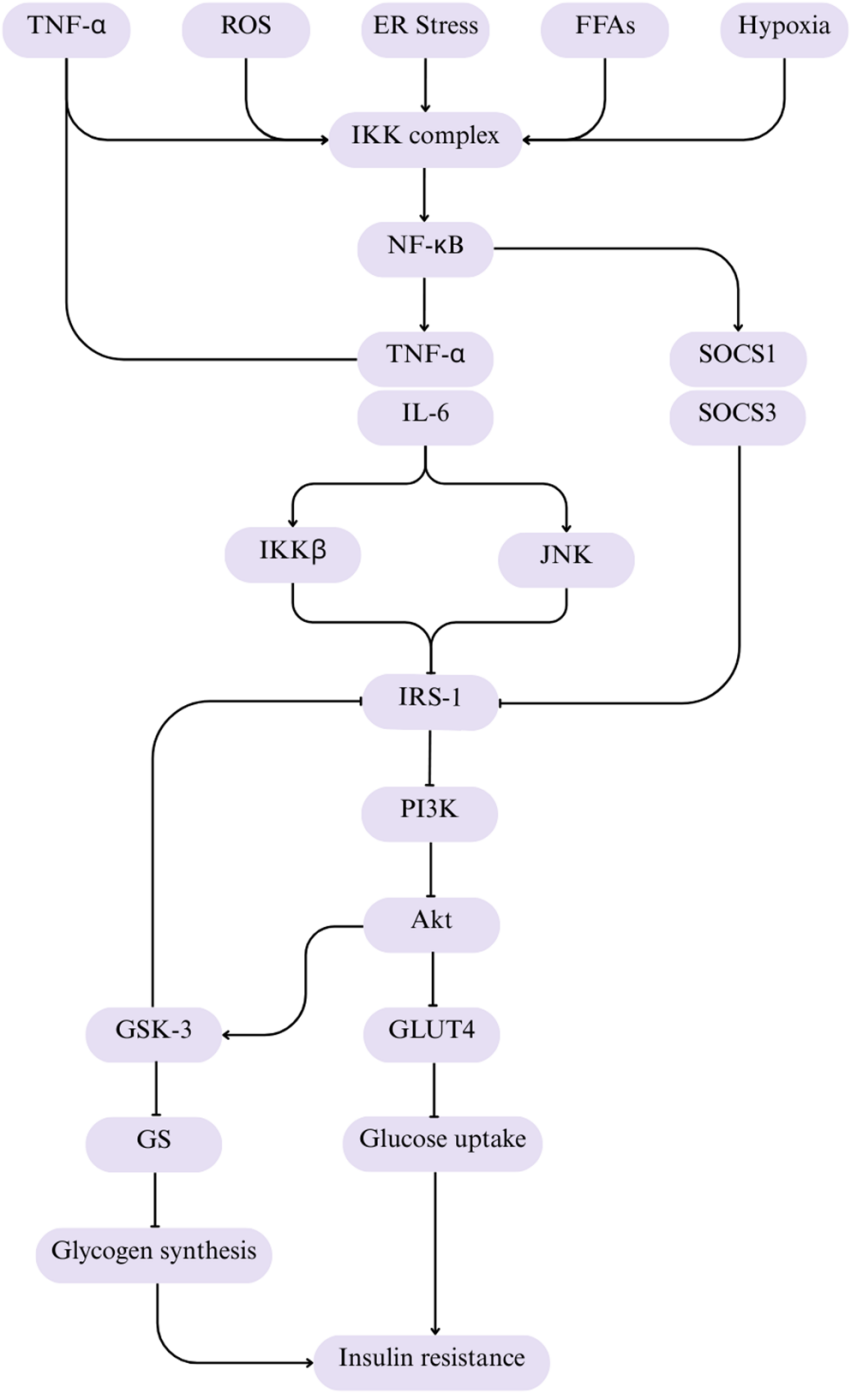
The Nuclear Factor-kappa B (NF-κB) pathway in developing insulin resistance. Inflammatory stimuli including TNF-α, ROS, ER stress, FFAs, and hypoxia activate the IKK complex, resulting in activation of NF-κB signalling. NF-κB induces the expression of pro-inflammatory cytokines such as TNF-α and IL-6, as well as SOCS1 and SOCS3. These mediators impair IRS-1 signalling through IKKβ- and JNK-dependent pathways. Disruption of IRS-1 signalling reduces PI3K and Akt activity, leading to decreased GLUT4 translocation, reduced glucose uptake, impaired glycogen synthesis due to dysregulation of GSK-3 activity, and ultimately the progression of insulin resistance. TNF-α, tumor necrosis factor alpha; ROS, reactive oxygen species; ER stress, endoplasmic reticulum stress; FFAs, free fatty acids; IKK, IκB kinase; NF-κB, nuclear factor-kappa B; IL-6, interleukin-6; IKKβ, inhibitory kappa B kinase beta; JNK, c-Jun N-terminal kinase; SOCS1, suppressor of cytokine signalling 1; SOCS3, suppressor of cytokine signalling 3; IRS-1, insulin receptor substrate 1; PI3K, phosphatidylinositol 3-kinase; Akt, protein kinase B; GLUT4, glucose transporter 4; GSK-3, glycogen synthase kinase-3; GS, glycogen synthase. Illustration created using Canva (https://www.canva.com)

The NF-κB pathway activates the transcription of pro-inflammatory genes, which causes the continuous production of pro-inflammatory cytokines such as TNF-α and IL-6^60^. These cytokines activate serine kinases, including inhibitory IKKβ and JNK [61]. They phosphorylate IRS-1 on serine residues [62], such as Ser^307^. The serine phosphorylation on IRS-1 inhibits tyrosine phosphorylation, which is crucial in insulin signalling. At the same time, the activated NF-κB pathway induces the expression of suppressors of cytokine signalling (SOCS), such as SOCS1 and SOCS3, which tend to bind to IRS-1, inhibiting the downstream signalling pathway and contributing to insulin resistance [61]. Inhibition of IRS-1 inactivates PI3K, which further inhibits Akt [63]. Akt inhibition reduces GLUT4 translocation. This condition reduces glucose uptake even in the presence of insulin. The cell does not respond to insulin, leading to insulin resistance and elevated blood sugar levels. Akt also activates GSK-3 [64]. This activation inhibits GS, which further inhibits glycogen synthesis [65]. Reduced glycogen levels slow glucose uptake even when insulin is present, leading to insulin resistance. The mechanism of NF-κB pathway is summarized in Fig. 3.

### Therapeutic approaches for obesity-induced insulin resistance

Therapeutic strategies for obesity-induced insulin resistance can be broadly classified into three categories: Plant-derived biofunctional compounds, lifestyle-based interventions and conventional pharmacological therapies.

#### Plant-derived biofunctional molecules

Natural polyphenols such as curcumin, resveratrol and epigallocatechin-3-gallate (EGCG) potentially help reduce obesity-induced insulin resistance [66]. Curcumin can be obtained from turmeric. Curcumin-loaded liposomes can reduce the production of inflammatory cytokines (TNF-α and IL-6) and improve antioxidant levels (superoxide dismutase), thus reducing inflammation and oxidative stress that will lead to insulin resistance [67]. Resveratrol is mainly extracted from the skins of red grapes and red wine [68]. It can activate the Akt pathway, which enhances glucose uptake via GLUT4 membrane translocation, further improving insulin sensitivity [69]. EGCG can be obtained from green tea. It treats insulin resistance by upregulating IRS-1 and activating AMPK, increasing glucose uptake [70]. At the same time, it inhibits pro-inflammatory cytokine secretion, restoring insulin sensitivity.

Other traditional herbal extracts, such as berberine and bitter melon, also contribute to insulin sensitivity. Berberine is an alkaloid extracted from *Coptis chinensis* [71]. It activates AMPK to improve glucose uptake and enhance insulin signalling transduction by reducing ER stress [72]. *Momordica charantia* L., also known as bitter melon, is used as a natural supplement to treat complications of obesity, including insulin resistance. It enhances lipid metabolism and glucose homeostasis by activating AMPK and PPARs, thereby improving insulin sensitivity in people living with obesity [73].

#### Lifestyle-based modifications

The practical solution for obesity-induced insulin resistance can start with a healthy diet. Study suggested the Mediterranean diet and low-caloric food, which is rich in whole grains, legumes, fruits, vegetables, healthy fats such as olive oil and omega-3 fatty acids improve insulin sensitivity [74]. Whole grains and legumes have a low glycemic index, which leads to a slower increase in blood sugar levels [75]. Mediterranean fruits and vegetables are rich in polyphenols, which can inhibit oxidative stress and inflammation pathways [76]. Omega-3 fatty acids help regulate blood sugar levels and lipid profiles while exhibiting anti-inflammatory and antioxidant properties [77].

Exercise also helps treat insulin resistance by improving metabolic health. A study found that exercising 5 days per week, with each session lasting 45 min and including an aerobic and a strength period, significantly reduces fasting and postprandial blood glucose levels [78]. Exercise also promotes FFAs oxidation, thus reducing lipotoxicity in muscles, enhancing insulin sensitivity. Another study reported that regular exercise helps in anti-inflammatory responses while promoting antioxidant defenses against oxidative stress to maintain normal mitochondrial functions [79] (Table 1).

**Table 1:** Mechanistic basis of metabolic dysfunction and targeted therapeutic strategies

Section	Metabolic consequences	Key mechanism	Clinical evidence	Therapeutic strategies	References
Adipocyte hypertrophy increases lipolysis	Release FFA	TLR2 & TLR4 → MyD88→ IKK→NF-κB	Adipose tissue in people living with obesity becomes inflamed, with increased secretion of TNFα. TNFα directly stimulates lipolysis, contributing to the enhanced FFA release.	*Conventional treatment*: liraglutide, dapagliflozin *Natural products*: berberine, bitter melon *Lifestyle changes*: exercise	[80], [81], [82]
Adipocyte hypertrophy leads to ectopic fat accumulation	Lipotoxicity induces inflammation and oxidative stress; Up-regulating pro-inflammatory cytokines and produce more ROS	*Cytokines*: TNFR1→IKK→NF-κB *ROS*: IKK→NF-κB	Increased adipocyte size is associated with hepatic and intramyocellular lipid accumulation in individuals with obesity and insulin resistance.	*Conventional treatment*: metformin, TZDs *Natural products*: curcumin, resveratrol *Lifestyle changes*: Mediterranean diet, exercise	[83], [84], [85], [86]
Adipocyte hypertrophy induces ER stress	Up-regulating pro-inflammatory cytokines	TNFR1→IKK→NF-κB	Adipose tissue biopsies from individuals with obesity showed that markers of endoplasmic reticulum stress are elevated and correlate with insulin resistance severity.	*Conventional treatment*: metformin *Natural products*: EGCG *Lifestyle changes*: Mediterranean diet	[83], [87], [88]
Adipocyte hypertrophy causes adipokine imbalance	Up-regulating pro-inflammatory cytokines; down-regulating anti-inflammatory cytokines	TNFR1→IKK→NF-κB	Individuals with obesity demonstrated elevated levels of TNF-α and IL-6.	*Conventional treatment*: TZDs, liraglutide *Natural products*: resveratrol, berberine *Lifestyle changes*: Mediterranean diet, exercise	[89], [90], [91]
Adipocyte hypertrophy silences HSP60 gene	Induce oxidative stress; produce more ROS	IKK→ NF-κB	Individuals with obesity exhibited the reduced expression of intracellular HSP60.	*Natural products*: curcumin, resveratrol *Lifestyle changes*: Mediterranean diet	[83], [92], [93]
Adipocyte hypoxia induces adipokine imbalance	Up-regulating pro-inflammatory cytokines	HIF-1α→RAGE→NF-κB	Hypoxia markers are elevated in adipose tissue of people with obesity, leading to altered adipokine secretion and impaired insulin sensitivity.	*Conventional treatment*: TZDs, liraglutide *Natural products*: resveratrol, berberine *Lifestyle changes*: Mediterranean diet, exercise	[83], [86], [89–91], [94]
Adipocyte hypoxia induces adipocyte necrosis	Up-regulating pro-inflammatory cytokines	TNFR1→IKK→NF-κB	Clinical studies have reported hypoxia within expanded adipose tissue depots. This localized hypoxia is correlated with increased adipocyte size and the presence of dead adipocytes.	*Natural products*: curcumin, EGCG *Lifestyle changes*: exercise	[86], [87], [95]
Chronic low-grade inflammation cause macrophage infiltration	Up-regulating pro-inflammatory cytokines; down-regulating anti-inflammatory cytokines	TNFR1→IKK→NF-κB	Increased macrophage infiltration and crown-like structures are reported in individuals with obesity.	*Conventional treatment*: TZDs *Natural products*: curcumin, resveratrol *Lifestyle changes*: Mediterranean diet, exercise	[83], [86], [89], [94], [95]
Oxidative stress caused by mitochondria dysfunction	ER stress produces more ROS	*ER stress*: UPR→ IRE1→PERK→ATF6 →TRAF2→IKK→NF-κB *ROS*: IKK→NF-κB	Individuals with obesity demonstrated impaired mitochondrial oxidative capacity and increased ROS production in skeletal muscle and adipose tissue.	*Conventional treatment*: metformin *Natural products*: EGCG *Lifestyle changes*: Mediterranean diet	[83], [96], [97]
Oxidative stress caused by NOX	Produce more ROS	IKK→NF-κB	Individuals with obesity exhibited elevated NOX expression and activity.	*Natural products*: EGCG, curcumin, berberine *Lifestyle changes*: Mediterranean diet	[83], [92], [96], [98]

#### Conventional Pharmacological treatments

**Fig. 4:**
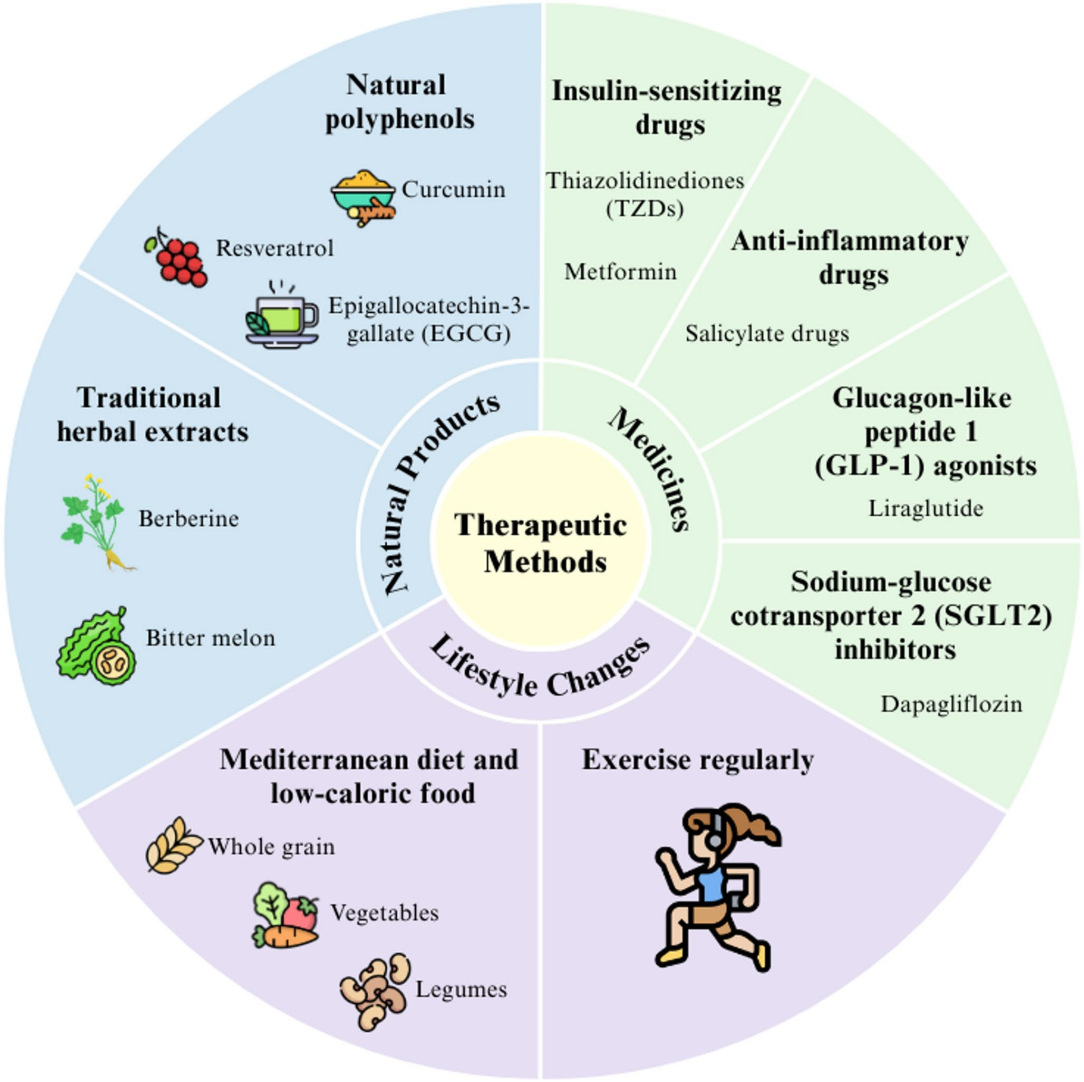
Schematic representation of Therapeutic methods for obesity-induced insulin resistance. Therapeutic approaches for obesity-induced insulin resistance are broadly categorized into natural products, pharmacological interventions, and lifestyle modifications. Natural products include polyphenols such as resveratrol, curcumin, and EGCG, as well as traditional herbal extracts, such as berberine and bitter melon, which exert antioxidant, anti-inflammatory, and insulin-sensitizing effects. Pharmacological treatments include insulin-sensitizing agents, anti-inflammatory drugs, GLP-1 receptor agonists, and SGLT2 inhibitors. Lifestyle interventions such as adherence to a Mediterranean diet, calorie restriction, and regular physical activity play a critical role in improving metabolic homeostasis and insulin sensitivity. EGCG, epigallocatechin gallate; GLP-1, glucagon-like peptide-1; SGLT2, sodium-glucose cotransporter 2. Illustration created using Canva (https://www.canva.com)

This subsection summarizes the major pharmacological classes used clinically to improve insulin sensitivity, with emphasis on their underlying mechanisms of action. The conventional pharmacological treatments refer to the drugs that are commonly used to improve insulin sensitivity, which can be divided based on their function, including insulin-sensitizing drugs, anti-inflammatory drugs, glucagon-like peptide 1 (GLP-1) agonists, and sodium-glucose cotransporter-2 (SGLT2) inhibitors (Figure. 4). Metformin is an insulin-sensitising drug that triggers the adenosine monophosphate (AMP)-activated protein kinase (AMPK) pathway. AMPK activation increases glucose uptake by promoting Akt phosphorylation and GLUT4 translocation, thus improving insulin signalling [99]. Metformin also downregulates the expression of RNA NONMMUT031874.2, further reducing SOCS3 expression and enhancing insulin sensitivity [100]. Thiazolidinediones (TZDs), for example, pioglitazone and rosiglitazone, is a class of peroxisome proliferator-activated receptor gamma (PPAR*γ)* agonists used to improve insulin sensitivity [101]. TZDs bind to PPAR-γ receptors, increase lipid storage capacity in adipose tissue and reduce lipotoxicity. TZDs also produce more adiponectin and less TNF-α and interleukin, reducing the inflammation response [102]. In addition, drugs containing salicylate compounds have anti-inflammatory properties. They inhibit TNF secretion, thereby blocking IKK activation and improving insulin action [103]. Furthermore, liraglutide, a GLP-1 agonist, treats insulin resistance by reducing macrophage accumulation, thereby reducing inflammation and superoxide production and, in turn, oxidative stress in the early stages of obesity [104]. Moreover, dapagliflozin is an SGLT2 inhibitor used in patients with insulin resistance to increase glucose excretion via urination. This is important to maintain a standard range of insulin required for specific glucose disposal rates, thereby improving insulin sensitivity [105]. In addition to lifestyle and pharmacological approaches, metabolic-bariatric surgery offers a highly effective intervention for insulin resistance by promoting sustained weight loss, reducing ectopic fat deposition, improving adipokine profiles, and enhancing insulin signalling, thereby providing long-term metabolic benefits for individuals living with obesity [106].

This review presents a novel integrative strategy to obesity-induced insulin resistance by combining conventional pharmaceutical therapies, natural products, and lifestyle modifications. Unlike prior reviews that focus on individual treatment approaches, our analysis highlights recent mechanistic insights, innovative molecular targets, and the relative effectiveness of multi-modal strategies, stressing practical implications for translational and personalized therapies. Furthermore, we analyze gender-, ethnic-, and lifestyle-specific characteristics, offering valuable insights for targeted therapeutics and addressing obesity-related insulin resistance issues across diverse populations.

### Future perspectives on insulin resistance targeting to prevent metabolic diseases

This section adopts a translational perspective to highlight emerging strategies that extend beyond current pharmacological and lifestyle interventions for obesity-induced insulin resistance. Building on the mechanistic evidence discussed earlier, these future directions focus on improving therapeutic precision, scalability, and long-term metabolic outcomes by targeting individual variability, leveraging digital technologies, and modulating disease-driving pathways at the genetic level. Together, these approaches address key limitations of existing treatments and offer promising avenues for treating insulin resistance-associated metabolic diseases.

#### Personalized medicine

Studies suggested future insights for insulin resistance targeting, which include developing personalized medicine, advancing digital health and exploring gene editing. Personalized medicine provides customized treatment plans to the patients, addressing the heterogeneity of insulin resistance based on their specific body conditions, such as genetic makeup, lifestyle factors, environmental influences and other health data [107]. Compared to the conventional one-size-fits-all approach, personalized medicine is more precise and yields better treatment outcomes. Pharmacogenomic insights provide a deeper understanding of the efficacy and tolerability of medications, such as metformin, based on individuals’ genetic profiles [108]. This provides a better drug selection while minimizing side effects, thereby enhancing treatment outcomes. For example, people with functional PRPF31, CPA6 and STAT3 genes improve the metformin response [109]. However, people with the variants T199I, T201M, A270S at the *SLC22A2* gene have a poor response to metformin, thus must consider other treatment options [110].

#### Digital health technologies and artificial intelligence (AI)

Advanced digital health technologies and artificial intelligence (AI) are emerging as tools to help manage insulin resistance and support predictive and preventive care. For example, *Healthy at Home* is a 12-week phone and short message service-based digital health coaching program on insulin resistance. The study found that this digital health coaching improved treatment outcomes for insulin resistance by providing personalized and scalable interventions to manage the metabolic disease through lifestyle and behavioural changes [111]. Besides, the digital twin technology is used to integrate mechanistic mathematical models for insulin resistance analysis across different timescales and physiological levels. It can predict treatment outcomes related to weight changes and insulin signalling, thereby providing precise management strategies for individuals [112].

#### Gene editing technologies

Gene editing holds significant promise in treating obesity-induced insulin resistance. For example, a previous study highlighted the use of clustered regularly interspaced short palindromic repeats-associated protein 9 (CRISPR-Cas9) and single guide ribonucleic acid (RNA) to target the overexpressed fatty acid-binding protein 4 (FABP4) gene [113]. Silencing of the FABP4 gene effectively suppresses obesity-induced inflammation, further reducing insulin resistance. A recent study successfully knocked out the nuclear receptor-interacting protein 1 (NRIP1) gene using CRISPR, thereby upregulating other genes highly expressed in brown adipocytes to improve glucose tolerance and insulin sensitivity [114]. Besides, the adeno-associated virus (AAV) mediated gene therapy offers a long-lasting therapeutic effect to address obesity-induced insulin resistance. AAV vectors are used to deliver genes, including bone morphogenetic protein 7 (BMP7) and fibroblast growth factor 21 (FGF21) to the target tissues in mice without significant side effects [113, 114]. These research studies are conducted on mice with obesity; thus, future research should focus on the safety for clinical applications in humans.

## Conclusion

Insulin resistance caused by obesity remains a major metabolic issue globally, driven by adipose tissue dysfunction, inflammation, and oxidative stress. The resultant metabolic imbalance impairs insulin signalling, leading to systemic insulin resistance. Current pharmaceutical therapies, such as metformin, TZDs, and GLP-1 agonists, enhance insulin sensitivity; however, personalized medicine and pharmacogenomics offer more tailored therapy options. Emerging technologies, including digital health solutions and gene editing, possess the capacity to transform the management of insulin resistance. Managing this intricate illness requires a multidisciplinary approach that integrates clinical, technical, and individualized solutions. Global implementation of these improvements is essential to alleviate obesity-related metabolic disorders and enhance long-term health outcomes.

## Data Availability

The datasets generated are available from the corresponding author on reasonable request.

## Declarations

### Data sharing

The datasets generated are available from the corresponding author on reasonable request.

## Glossary

Adeno-Associated Virus: A small virus used as a vector in gene therapy due to its ability to deliver genetic material safely.
Artificial Intelligence: Computer-based systems that simulate human intelligence for tasks such as diagnosis, prediction, and data analysis.
Protein Kinase B: A central kinase in insulin signalling that regulates glucose uptake, metabolism, and cell survival.
Adenosine Monophosphate: A nucleotide that plays a role in energy metabolism and regulation of cellular energy balance.
AMP-Activated Protein Kinase: An energy sensor that regulates glucose and lipid metabolism, improving insulin sensitivity.
Body Mass Index: A measure of body fat based on weight and height, used to classify overweight and obesity.
Endoplasmic Reticulum: A cellular organelle involved in protein folding, lipid synthesis, and calcium storage.
Electron Transport Chain: A mitochondrial system that produces ATP but also generates reactive oxygen species (ROS).
Free Fatty Acids: Lipid molecules released from adipose tissue that, in excess, cause lipotoxicity and insulin resistance.
Inflammatory Bowel Disease: A chronic inflammatory condition of the gastrointestinal tract.
Metabolic Dysfunction-Associated Steatotic Liver Disease: A liver disease characterized by fat accumulation and insulin resistance.
Non-Alcoholic Fatty Liver Disease: A liver disorder characterized by fat accumulation without alcohol use.
Non-Communicable Diseases: Chronic diseases like diabetes, cardiovascular disease, and obesity.
Reactive Oxygen Species: Chemically reactive molecules causing oxidative stress and cell damage.
